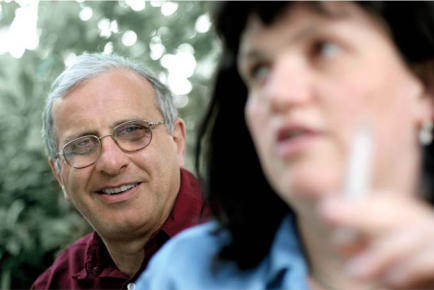# Neurology: Dementia and Secondhand Smoke

**DOI:** 10.1289/ehp.115-a401a

**Published:** 2007-08

**Authors:** Julia R. Barrett

Secondhand smoke is known to be associated with cardiovascular disease, which in turn is a known risk factor for dementia, but little research has examined the latter end point with regard to secondhand smoke. Research presented at the 28 April–5 May 2007 annual meeting of the American Academy of Neurology now suggests that increased risk of Alzheimer disease and other forms of dementia may well belong on the long list of potential health effects from chronic exposure to secondhand smoke.

“When we started this study, . . . we knew of the likely pathway through cardiovascular disease, but we were also interested in an independent pathway from secondhand smoke [directly] to dementia,” says principal investigator Thaddeus Haight, a senior statistician at the University of California, Berkeley. That direct pathway did not bear out, but the team did make a new discovery regarding the cardiovascular link.

Haight and his colleagues analyzed health data for elders with and without cardiovascular disease that had been collected through the Cardiovascular Health Study, a national study of cardiovascular disease risk factors in adults older than 65. Of the 3,602 participants who had been evaluated for dementia, 985 had no history of cardiovascular disease or symptoms of dementia and had never smoked, and 495 reported an average of nearly 28 years of secondhand smoke exposure.

The group most highly exposed to secondhand smoke—those with a lifetime exposure of more than 30 years—had a 30% greater risk for developing dementia compared with the no-exposure group. Within the highly exposed group, people with subclinical cardiovascular disease (defined as narrowing of the carotid artery) had an even higher risk, nearly 2.5 times that of the no-exposure group. “There weren’t really any independent effects due to secondhand smoke exposure alone, but there were effects through a pathway other than clinical cardiovascular disease,” says Haight. “In people with indications of subclinical disease, . . . the greater the exposure to secondhand smoke, the more elevated the risk of dementia.”

“These results are definitely not surprising; they are completely in line with what we know about heart health and brain health—that both are extremely interconnected,” says Maria Carrillo, director of medical and scientific relations for the Alzheimer’s Association national office. “Anything that makes it more difficult for your heart to pump blood through your body will ultimately affect how your body pumps blood into the brain. That compromised brain blood volume really makes a difference after years of exposure.”

Haight and his colleagues are currently working to confirm their findings. He says, “What would be interesting from this work that we’ve done so far is to extend it to look at the risk of dementia in those with a history of secondhand smoke exposure who have diabetes or hypertension.”

## Figures and Tables

**Figure:**